# In Vivo Assessment and Monitoring of Burn Wounds Using a Handheld Terahertz Hyperspectral Scanner

**DOI:** 10.1002/adpr.202100095

**Published:** 2022-01-17

**Authors:** Omar B. Osman, Zachery B. Harris, Juin W. Zhou, Mahmoud E. Khani, Adam J. Singer, M. Hassan Arbab

**Affiliations:** Department of Biomedical Engineering, Stony Brook University, Stony Brook, NY 11794, USA; Department of Biomedical Engineering, Stony Brook University, Stony Brook, NY 11794, USA; Department of Biomedical Engineering, Stony Brook University, Stony Brook, NY 11794, USA; Department of Biomedical Engineering, Stony Brook University, Stony Brook, NY 11794, USA; Department of Emergency Medicine, Renaissance School of Medicine at Stony Brook University, 101 Nicolls Rd., Stony Brook, NY 11794, USA; Department of Biomedical Engineering, Stony Brook University, Stony Brook, NY 11794, USA

**Keywords:** burn imaging, handheld THz-TDS scanner, histology, partial-thickness burn characterization, skin tissue hyperspectral imaging, terahertz time-domain spectroscopy

## Abstract

The accuracy of clinical assessment techniques in diagnosing partial-thickness burn injuries has remained as low as 50–76%. Depending on the burn depth and environmental factors in the wound, such as reactive oxygen species, inflammation, and autophagy, partial-thickness burns can heal spontaneously or require surgical intervention. Herein, it is demonstrated that terahertz time-domain spectral imaging (THz-TDSI) is a promising tool for in vivo quantitative assessment and monitoring of partial-thickness burn injuries in large animals. We used a novel handheld THz-TDSI scanner to characterize burn injuries in a porcine scald model with histopathological controls. Statistical analysis (n= 40) indicates that the THz-TDSI modality can accurately differentiate between partial-thickness and full-thickness burn injuries (1-way ANOVA, p< 0.05). THz-TDSI has the potential to improve burn care outcomes by helping surgeons in making objective decisions for early excision of the wound.

## Graphical Abstract

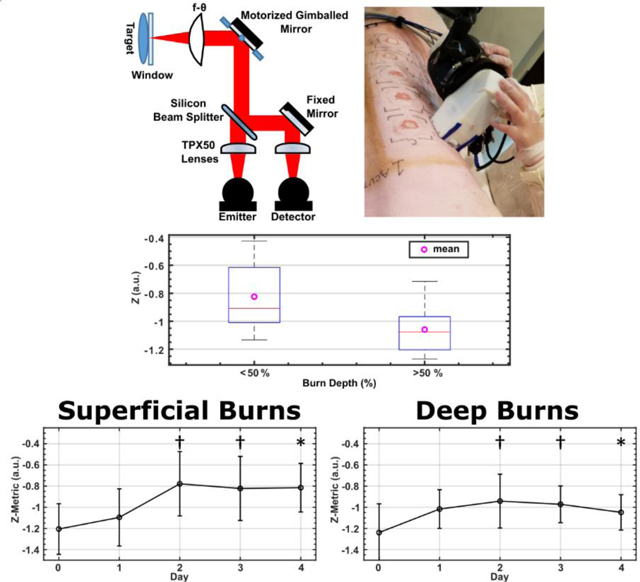

The authors use a novel Terahertz Portable Handheld Spectral Reflection (PHASR) Scanner to characterize burn injuries in a longitudinal porcine scald model. A terahertz hyperspectral analysis is presented that can accurately differentiate between burns and monitor their progression during the healing process.

## 1. Introduction

Acute burn injuries accounted for ≈489 000 visits to emergency departments in the United States, in 2017.^[1]^ During triage, burn injuries are clinically assessed based on a subjective method that relies on the visual and tactile inspection of the injury. A physician will determine the course of the treatment based on the perceived depth of the thermal damage. According to the clinical definition, superficial burns affect only the epidermis layer of the skin and will heal spontaneously by the reepithelialization process. Full-thickness burns, however, affect the entire depth of the dermis. Additionally, full-thickness burns can result in hypertrophic or contracted scarring and will require surgical intervention (i.e. excision and grafting).^[2]^ Partial-thickness burns, which affect the epidermis, papillary region, and reticular layer, are challenging to assess because they may heal spontaneously or progress into deeper burns. Wound conversion in partial-thickness burns depends on multiple factors, including perfusion,^[3]^ reactive oxygen species,^[4]^ and mechanisms such as autophagy and inflammation.^[4]^ The accuracy of the clinical evaluation for determining whether an indeterminate or deep partial-thickness burn will progress into a full-thickness burn has been shown to be as low as 50–76%.^[5–7]^ This low-accuracy rate often results in delaying clinical diagnosis until the burn “declares its nature.” In other words, the diagnosis must wait until the burn reaches its maximum depth, which usually occurs after a few days post-burn. On the other hand, early excision and grafting of full thickness burns results in overall better patient outcomes.^[8–10]^ Therefore, early and accurate determination of partial-thickness burn injuries can improve wound healing and reduce the recovery time and financial burden of these injuries.

Terahertz time-domain spectroscopy (THz-TDS) is a coherent broadband spectral imaging technique in which a pulsed terahertz (THz) electric field can be reflected from a sample and measured in the time-domain (TD) with subpicosecond resolution. THz radiation, defined by electromagnetic waves with frequencies between 0.1 and 10 THz, has piqued the interest of biomedical researchers, especially for in vivo applications in skin^[11,12]^ and corneal^[13–16]^ imaging, due to the high sensitivity of THz waves to absorption by the water content of tissue. The broad and strong absorption of water at the THz frequencies is due to the collective intermolecular motions of bound and free water molecules.^[17]^ Additionally, thanks to the longer wavelengths of the radiation, THz modalities are less prone to electromagnetic scattering at the cellular level, as compared with other optical imaging techniques.^[18,19]^ Also, many biomolecules in their polycrystalline form have fingerprint spectral resonances in the THz wavelengths.^[18,19]^ Among these notable features, it is the sensitivity of the THz-TDS technique to small perturbations in the tissue hydration that makes it a promising imaging modality for diagnosis of burn injuries.^[20,21]^ Furthermore, THz radiation has very low photon energy levels (few meV) and, therefore, is nonionizing and safe for use in humans and animals. Previous safety studies have determined that there are no significant negative in vivo effects due to exposure to THz radiation, especially at the power levels achievable using photoconductive antenna (PCA) emitters.^[22]^ Additional studies have observed thermal and nonthermal effects as the result of much higher power levels of the THz radiation and at longer exposure durations.^[22–24]^ The American National Standards Institute (ANSI), which provides guidelines for safe use of lasers in industrial, military, medical and other applications, has determined that the Maximum Permissible Exposure Limit of skin to <1 mm wave radiation (>0.3 THz frequency, or >1.2 meV photon energy) is 0.1 W cm^2^ for 10 s or less.^[25]^ The THz time-domain spectral imaging (THz-TDSI) scanner introduced in this study is well within this safety standard by using a pulsed system having a 0.02% duty cycle and <0.37 mW cm^−2^ intensity for less than 0.3 s scan time per pixel. Therefore, the total exposure time of each pixel is 0.06 ms.

Several research groups have approached using THz imaging to measure burn injuries, in both ex vivo^[20,26]^ and in vivo models.^[21,27–29]^ Upon the infliction of a burn injury, certain physiological events occur that increase the tissue water content, such as increased capillary permeability, increased hydrostatic pressure, and formation of edema.^[30]^ THz studies using in vivo rat models showed that the THz spectra of burns exhibited increased reflectivity at 72 h postburn.^[20,21,27,31]^ Additionally, THz-TDS studies in rodent models further elucidated a secondary signal contrast mechanism by correlating broadband THz spectra with the density of structures within the skin.^[20,21,31]^ Recently, we demonstrated that similar signal contrast mechanisms exist in an in vivo porcine model.^[32]^ However, our instrument lacked imaging capability and was susceptible to motion artifacts, e.g., due to animal respiration during longer data acquisition times. Moreover, Suen et al. have demonstrated a practical benefit of using THz radiation over infrared wavelengths because of its ability for transmission through typical burn wound topical ointments and wound dressings with little attenuation.^[33]^ The high transmissivity of burn ointments suggests that THz radiation has the potential to monitor burn injuries throughout the healing process without disturbing the wound dressing. However, the use of THz modalities for longitudinal monitoring of burn wound healing has not been demonstrated to this date.

Another practical consideration for the utility of the THz-TDS modality is its form factor. Most THz-TDS systems require a sample to be raster scanned around a fixed optical focus, which will not be suitable outside research laboratories or for applications involving large samples. To address this issue, a few research groups have developed compact and handheld THz devices. For example, a handheld battery-powered single-point THz spectroscopy device was developed for non-destructive testing applications.^[34]^ This device was also used for measuring the dielectric response of the human skin.^[35]^ A handheld single-line scanner was designed for imaging malignant breast tumors; however, it was restricted to scanning along a single axis with a 2 mm by 15 mm field-of-view (FOV).^[36]^ In contrast to these instruments, we have designed and built a handheld, fiber-coupled, 2D THz-TDSI scanner with a FOV of 12 19mm^2^, and have tested its utility in an in vivo porcine burn study using THz radiation.^[37,38]^

In this article, we present, for the first time, the application of our handheld portable THz scanner in an acute in vivo porcine scald study to evaluate the accuracy of the THz-TDSI modality in assessing burn injuries. Previous porcine scald studies used a pseudo-scald device by wrapping a plastic film around a bottomless glass bottle that was filled with hot water to conform to the surface of the skin.^[39]^ In addition to our prior reporting,^[32]^ other groups have also observed that this model results in certain burn depth variability between the anatomical locations, as well as between the center and the edges of each burn.^[39,40]^ This variability is because good contact between the skin and the heat source may be obstructed by skin surface heterogeneities and air pockets.^[39,40]^ Here, we address this concern by inducing the burns with a scalding device that allows hot water to be in direct contact with the skin.^[40]^ Furthermore, our previous work using a fiber-coupled commercial THz setup in a porcine burn model was restricted to single-point spectroscopic measurements.^[32]^ Here, we have developed the first handheld, alignment-free, and easily deployable THz-TDSI scanner for diagnostic mapping of the burn severity in large mammals. Also, previous studies were limited to a single assessment of the burns immediately after the injury and therefore were unable to monitor the change in the burn severity by tracking its THz spectroscopic response over time.^[32]^ In this work, we monitor the THz hyperspectral signatures of the burns over several days and during the peak of the inflammatory response. We will show that our THz-TDSI scanner can demarcate burned tissue using the integral of the spectral amplitude reflectivity. Also, we will show that using the dual-band hyperspectral slopes of the decon-volved THz amplitude, THz-TDSI can differentiate burns of different severities against histological assessments as the gold standard (*p* < 0.05). Finally, we will show the application of the same hyperspectral parameters in monitoring the change in the THz signatures and healing progression over the first 5 days post burn.

## 2. Results and Discussion

### 2.1 Hyperspectral Analysis

**Figure 1.**
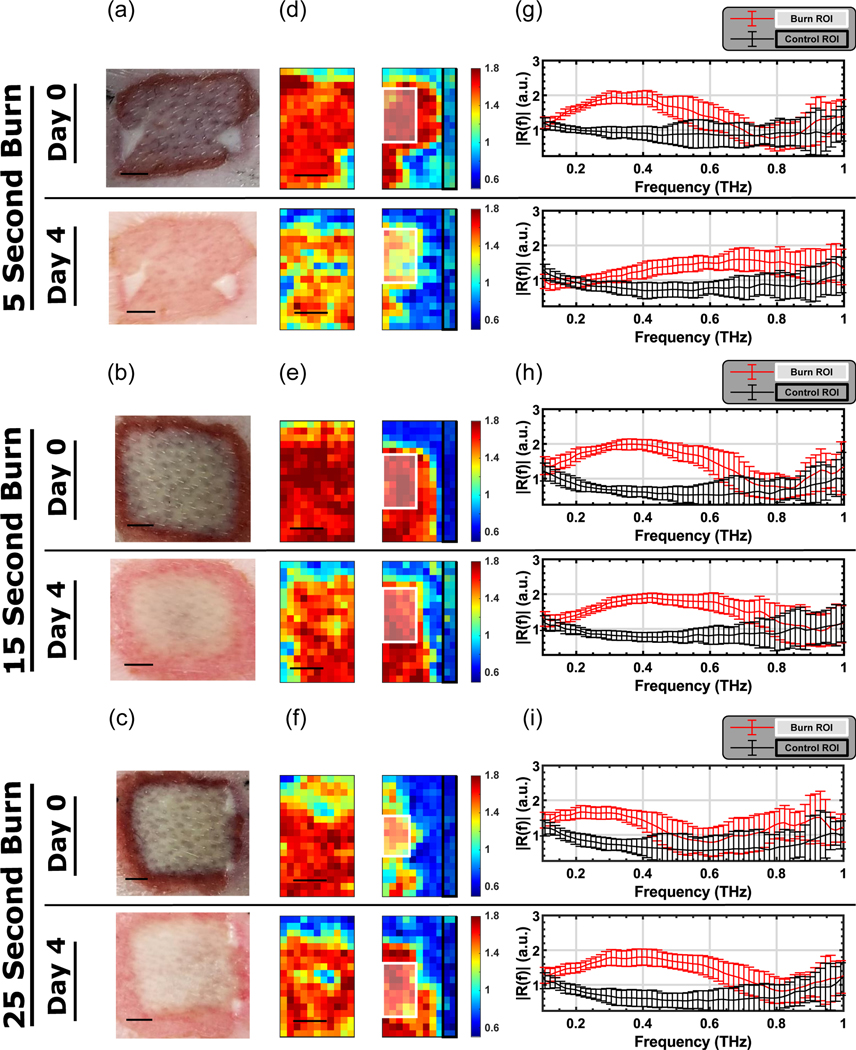
a–c) Visual images, d–f) THz images, and g–i) mean of the deconvolved THz spectra are shown for representative tissue samples from each burn condition on Days 0 and 4. The quantity shown in the color bar is SA. In (g–i), the red traces and red error bars (mean ± SD) correspond to the white ROIs drawn in the burned tissue in (d–f), while the black traces and black error bars (mean ± SD) correspond to the black ROIs drawn in the healthy skin regions. (Scale bar = 5 mm).

Figure 1 shows the visual and THz images of representative burns, along with the deconvolved reflectivity, *R(f)*, from the corresponding regions of interest (ROIs) for both burn and perilesional (healthy) skin samples. Due to the size of the wounds (20×20mm^2^) and the FOV (12×19mm^2^), we collected two THz images to cover the burn and perilesional skin areas. Pseudo-colored THz images were created by integrating *R(f)* in each pixel between the lower and upper frequency bins, f_lower_ and f_upper_, respectively. These bins were defined as the frequencies at which the amplitude of the reflected THz field, before deconvolution, were within −10.5 dB of its maximum in the lower (f_lower_) and higher (f_upper_) frequencies. The value for −10.5 dB was chosen to ensure that a consistent bandwidth could be used for the analysis without approaching the noise floor. The integral of the deconvolved reflectivity, R(f), between these frequency bins was then normalized by the number of frequency bins, *N*_bins_, to calculate the Spectral Area, *SA*, given by

(1) $$SA=\frac{1}{N_{\text{bins}}}\overset{f_{\text{upper}}}{\underset{f_{\text{lower}}}{\int }}R(f)df$$

The hyperspectral parameter SA was used as the color map in pseudocolored THz images in Figure 1d–f. These figures show that we can clearly demarcate the burn wound from healthy tissue using only the spectral area under the reflectivity curves, SA. Further, the contrast in the THz images mimicked the spatial features in the visual image. This allowed us to identify and create ROIs within the burned (white box) and healthy (black box) samples in Figure 1d–f. The reflectivity spectra shown in Figure 1g–i represent the mean spectra in the burn and healthy (control) ROIs in Figure 1d–f. It can be seen that the burn tissue (red trace and error bar) has higher reflectivity in our usable bandwidth when compared with the healthy tissue (black trace and error bar) for all burn conditions.

Results in Figure 1g–i show a qualitative difference between the superficial conditions (5 s heat exposure) and the deeper burn groups (e.g., the 25 s heat exposure). This contrast is particularly evident on Day 4 postburn, by which time the reflectivity of the superficial group has changed significantly whereas it has remained unchanged for the deeper burns. It should be noted that the absorption coefficient of the skin is relatively high, which limits the penetration depth of the THz light.^[35]^ Depending on the frequency and the anatomical location, the penetration depth in healthy skin is between 0.1 and <1 mm.^[41]^ We expect the penetration depth for burned skin to be comparable to that of the healthy skin. The thickness of the dermis layer is typically between 1 and 3 mm. Therefore, the THz light will not penetrate to the full thickness of the injured region. However, since the heat is applied from the outermost layer of the skin and propagates axially towards the subcutaneous tissue, the outermost area of the skin will experience the most thermal damage. Consequently, we expect to see the most spectral change in the area with the most damage. In other words, our results in Figure 1 suggest that although the THz light cannot reach the full-thickness regions, the spectral parameters of the more superficial layers can serve as a surrogate for the depth of the injury.

### 2.2. Statistical Analysis

**Figure 2.**
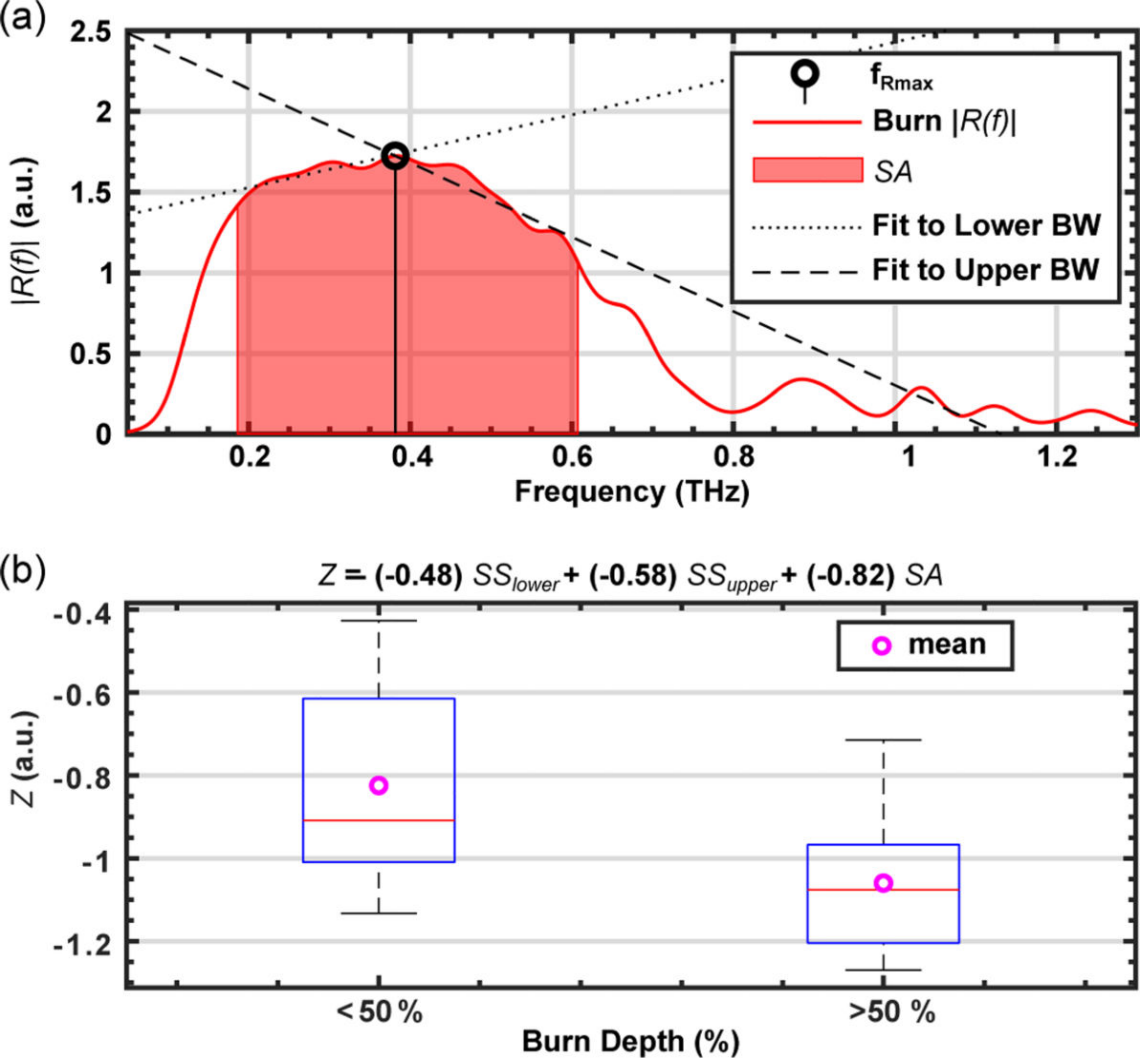
a) A representative THz amplitude reflectivity, *R(f)*, is plotted as an example to demonstrate the calculation of spectral slopes, *SS*, and spectral area, *SA*. The red area shows *SA* within the *f*_lower_ and *f*_upper_ frequencies. The dotted line shows the linear fit between *f*_lower_ and *f*_Rmax_ to calculate *SS*_lower_. The dashed line shows the linear fit between *f*_Rmax_ and *f*_upper_ to calculate *SS*_upper_. b) The ability of the Z parameter to differentiate between burn wounds is statistically tested (1-way ANOVA, *p* = 0.0016) against histological assessment of the burn depth. The THz measurements and biopsies were both obtained on Day 4 postburn. The burns were grouped into two categories: those with depth of damage greater or less than 50% of the dermis layer.

The SA parameter alone did not show significant differences between burn severities when compared with histological assessment. Therefore, we calculated the spectral slope, *SS*, of *R(f)* to capture the change in the THz reflectivity as a function of frequency. *SS* is defined as a dual-band hyperspectral parameter consisting of *SS*_upper_ and *SS*_lower_. The values for *SS*_upper_ and *SS*_lower_ are computed by calculating the slope of the linear fit to *R(f)* from *f*_Rmax_ to *f*_upper_, and from *f*_lower_ to *f*_Rmax_, respectively. *f*_Rmax_ is defined as the frequency bin where the amplitude of *R(f)* is the largest. This computation was performed for each pixel within a 5×8 pixel ROI in the burned region of each THz image. An example of the calculation of SS for a single pixel is shown in Figure 2a. The red shaded area shows the spectral range between *f*_lower_ and *f*_upper_, and represents the *SA* parameter defined in Equation (1), above. The dashed line shows the linear fit to the spectral amplitude curve between *f*_Rmax_ and *f*_upper_, which is then used for calculation of the *SS*_upper_ parameter. The dotted line shows the same fit between *f*_lower_ and *f*_Rmax_, which results in derivation of the *SS*_lower_. In certain examples, where tissue reflectivity was monotonically increasing or decreasing, as in Figure 1g, the values of *SS*_lower_ and *SS*_upper_ would be similar.

Our previous work using in vivo rodent^[21]^ and porcine^[32]^ models revealed that the THz spectral slopes contain valuable hyperspectral information. Because *SA* is calculated as the sum across a frequency range, this spectroscopic parameter is inherently less sensitive to frequency dependent behavior. The hyperspectral parameter SS, however, allows us to quantify frequency dependent changes in each ROI. One example of frequency-dependent behavior is electromagnetic scattering, which is dependent on the wavelength and the particle sizes,^[42–45]^ and is likely one of the sources of signal contrast in the *SS* parameters. Other sources of signal contrast include change in the complex refractive index of the sample as a result of the thermal energy. Therefore, the differences in the *SS* parameters between burns of varying severity may suggest that physiological and structural changes in the skin constituents postburn can serve as a source of imaging signal contrast. It should be noted that the THz wavelengths (3 mm–10 μm) are similar in size to some of these adnexal structures.

To utilize both the SA and the dual-band SS parameters in classifying burn injuries, we defined and optimized a combined hyperspectral parameter, Z, given by

(2) $$Z=a*SS_{\text{lower}}+b*SS_{\text{upper}}+c*SA$$

where *a*, *b*, and *c* are weighting coefficients for the *SS*_lower_, *SS*_upper_, and *SA*, respectively. Further, *a*, *b*, and *c* were optimized between −1 and 1 to maximize the separation between burn groups, which resulted in the following values: *a* = −0.48, *b* = −0.58, and *c* = −0.82. The optimization was performed via a brute-force search algorithm. The boxplot in Figure 2b shows the results of a one-way ANOVA test (*n* = 40) indicating statistical significance (*p* = 0.0016) in distinguishing burns with depth of damage greater than or less than 50% of the dermis layer using THz and histological data obtained on Day 4. Each sample in the statistical assessment was calculated by averaging the *SS*_lower_, *SS*_upper_, and *SA* over a 5×8 ROI within each burn sample image and following the preprocessing steps described in Section 4.4. Slight differences in the scanner placement on each day resulted in variations in the available burn area from each image. As a result, we used a consistently sized ROI (5×8 pixels) for all burns in the statistical analysis. Also, alternative hyperspectral algorithms, such as those based on the principal component analysis,^[46]^ may be able to capture similar physical phenomena in burn diagnosis.

When adnexal structures are destroyed in a full-thickness wound, re-epithelialization can only occur from the wound margins.^[47]^ In contrast, new epithelium in partial-thickness wounds originate from the vicinity of hair follicles and apocrine glands in swine, and from pilosebaceous units and apocrine sweat glands in humans.^[47,48]^ Considering the major role that adnexal structures play in the wound healing process, utilizing them as a contrast mechanism, by means of the *SS* parameters in THz-TDSI, provides a valuable window to monitor burn wound healing.

### 2.3. Burn Monitoring

**Figure 3.**
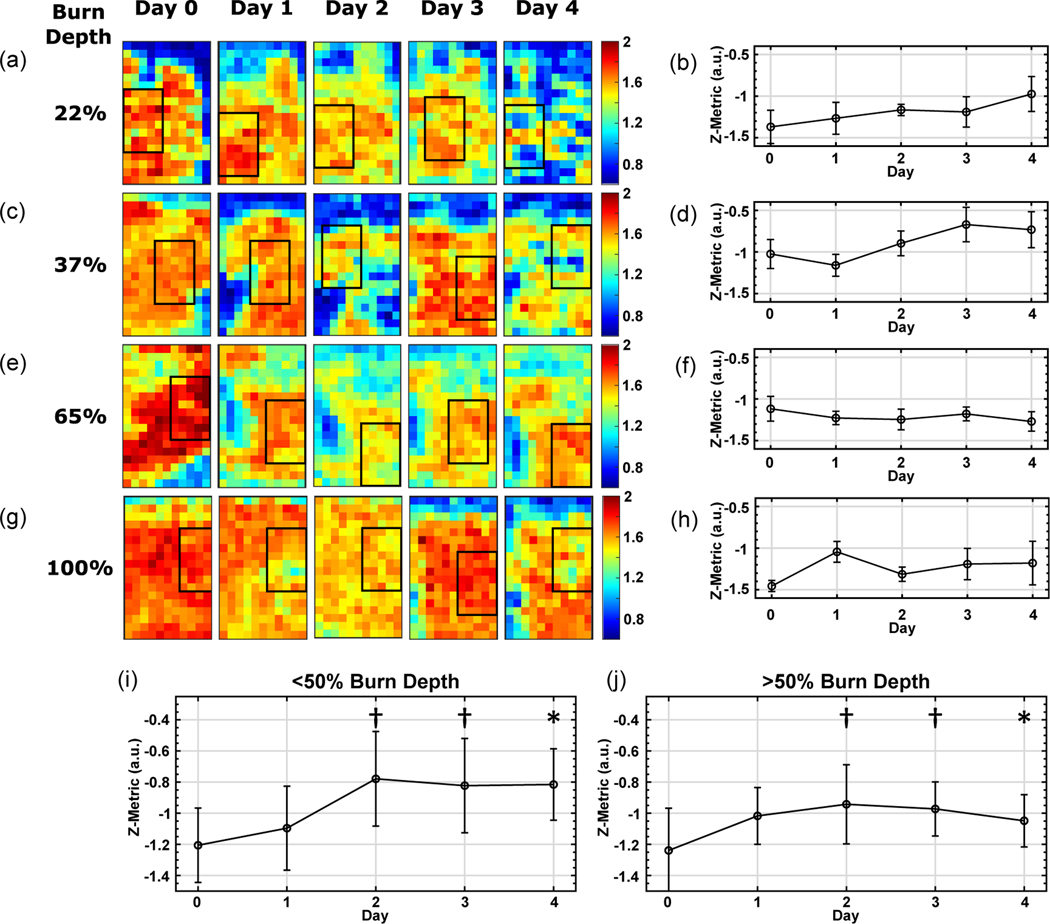
The daily longitudinal monitoring of the spectral area, SA, and the Z-parameter values (mean ± SD in the ROI subsets) are shown for four representative burn injuries: a,b) superficial partial thickness injury with 22% burn depth, c,d) superficial partial-thickness injury with 37% burn depth, e,f) deep-partial thickness injury with 65% burn depth, and g,h) full thickness injury with 100% burn depth. ROIs in (a, c, e, g) were drawn to be in the same general area as the previous day. Pixel size is 1 × 1 mm^2^. By including the THz data from all burns in this study, a trend similar to representative examples in (a-h) was observed. The Z parameter increased over time in burns with depth of damage less than 50% of the total dermal thickness (i), but remained relatively constant for burns with damage greater than 50% of the total dermal thickness (j). A one-way ANOVA between the <50% and >50% groups shows that *p* < 0.05 on Day 4 (represented by *) and *p* < 0.1 on Days 3 and 2 (represented by †).

In addition to differentiating between burns with depth of damage greater than and less than 50% of the total dermal thickness, we used the THz-TDSI scanner to monitor the wound progression over the course of the acute study. We used the same *a, b*, and *c* coefficients, which were optimized for the hyperspectral Z parameters on Day 4, to calculate the *Z*-value for the THz measurements obtained on Days 0 to 3. Figure 3a–d shows a longitudinal analysis of the THz hyperspectral parameters for two representative superficial partial-thickness burns, whose depths of dermal damage were 22% and 37%, respectively. In comparison, two representative deeper burns are also shown in Figure 3e–h, each having an injury depth of 65% and 100%. Figure 3a,c,e,g show the example locations, where the ROIs were marked on each day. To ensure an unbiased comparison between measurement days and to account for differences in the ROIs, we created 5 randomized subsets of 8 pixels within the ROI in each image. The standard deviation between the subsets is shown as the error bars in Figure 3b,d,f,h. Figure 3b,d shows a marked increase in the Z parameter for the two representative superficial partial-thickness burns, whereas it can be seen in Figure 3f,h that the same parameter remains relatively unchanged during the same observation period.

By including the THz data from all burns in this study, a trend similar to the representative examples in Figure 3a–h was observed. As shown in Figure 3i, the Z parameter increased over time in all burns with damage less than 50% depth of the total dermal thickness. Conversely, as it can be seen in Figure 3j, the Z parameter remained relatively constant for burns with damage greater than 50% depth of the total dermal thickness. A one-way ANOVA test between the burn groups with damage <50% and >50% of the dermal thickness shows that the difference in the *Z* parameter was statistically significant (*p* < 0.05) on Day 4, while it was found that *p* < 0.1 on Days 2 and 3. A similar phenomenon is observed in laser Doppler imaging, where perfusion increases in superficial burns, but remains limited in deeper burns.^[49]^ Compromised perfusion is a result of destroyed and damaged microvasculature postburn and is an important factor in the wound healing process.

## 3. Conclusion

In this work, we used a handheld THz-TDSI scanner to characterize burn injuries in an acute in vivo porcine scald model. The scanner was designed and built by our research team and included a custom-fabricated *f*-*θ* lens for 2D hyperspectral THz imaging. We showed that, by using the spectral area under the reflectivity curves, we could demarcate burned and healthy tissue zones. Utilizing the dual-band spectral slopes in the hyperspectral *Z* parameter allowed for differentiating between scald burns with damage greater or less than 50% of the total dermal thickness as determined by histological assessment. The use of the spectral area and the spectral slopes of the terahertz electric field to distinguish the different severities of burns suggests that the energy loss due to the electromagnetic scattering from skin constituents may play a role as an additional source of signal contrast to quantitatively assess burn injuries. Future modeling and experimental studies are needed to quantify the relative contribution of various sources of signal contrast in the THz-TDS measurements of skin samples. Finally, we showed the utility of our THz-TDSI scanner to differentiate and monitor the progression of the severity of the injury in partial-thickness burns over the course of 4 days after thermal insult. We showed that the hyperspectral Z parameter increased over time in burns with depth of injury less than 50% of the dermis layer, whereas it remained nearly unchanged in deeper burns. Further in vivo studies will include clinical burn wound healing endpoints, improved image co-registration and handheld THz instrumentation hardware to cover a larger FOV, faster scan rate, and enhanced image processing algorithms.

## 4. Experimental Section

### Study Protocol:

The experimental protocol used in this study was reviewed and approved by the Institutional Animal Care and Use Committee at the Stony Brook University. The animal model was based on female Landrace pigs that were 12-weeks of age and weighed ≈25–30 kg. Prior to inducing the burns on Day 0, the animal was sedated through an intramuscular (IM) injection of a preanesthetic cocktail consisting of ketamine (20 mg kg^−1^), xylazine (2.2 mg kg^−1^), acepromazine (0.1 mg kg^−1^), and atropine (0.02 mg kg^−1^), and then anesthetized with a continuous flow of 0.5–5% isoflurane. The pig skin was washed and the hair on its back was removed by trimming and shaving. A stencil was used to mark the locations for burn induction and guide the tattoo margins for tissue identification and image coregistration. Following burn induction, buprenorphine (0.005–0.02 mg kg^−1^) was administered IM and a 72-h transdermal fentanyl patch (50 μg kg^−1^) was placed proximal to the tail. Additionally, tattoo borders were placed to note the exact location of each burn. These corner tattoos acted as guides for the placement of the handheld scanner and ensured that the device was placed at the same area every day during the imaging studies. Following the tattooing procedure, the burns were debrided by gently scraping the burn with the blunt end of forceps, consistent with routine debridement of wounds in clinical burn care.

The animal was kept on isoflurane throughout the imaging process and monitored by the veterinary staff of the Division of Laboratory Animal Research at Stony Brook University. After imaging, the burns were bandaged by applying triple antibiotic ointment to the individual injuries and covering the wounds with a transparent Tegaderm sheet (3M, Saint Paul, MN, USA). The midsection of the pig was then wrapped with flexible gauze bandage and adhesive Tensoplast (BSN Medical, Hamburg, Germany). On Days 1 to 4, prior to imaging, the animal was sedated and anesthetized with the same procedure as Day 0. After imaging on Day 4, the entire burn and perilesional tissue was collected for histological assessment and the animal was euthanized through intravenous administration of Fatal Plus (100 mg kg^−1^).

**Figure 4.**
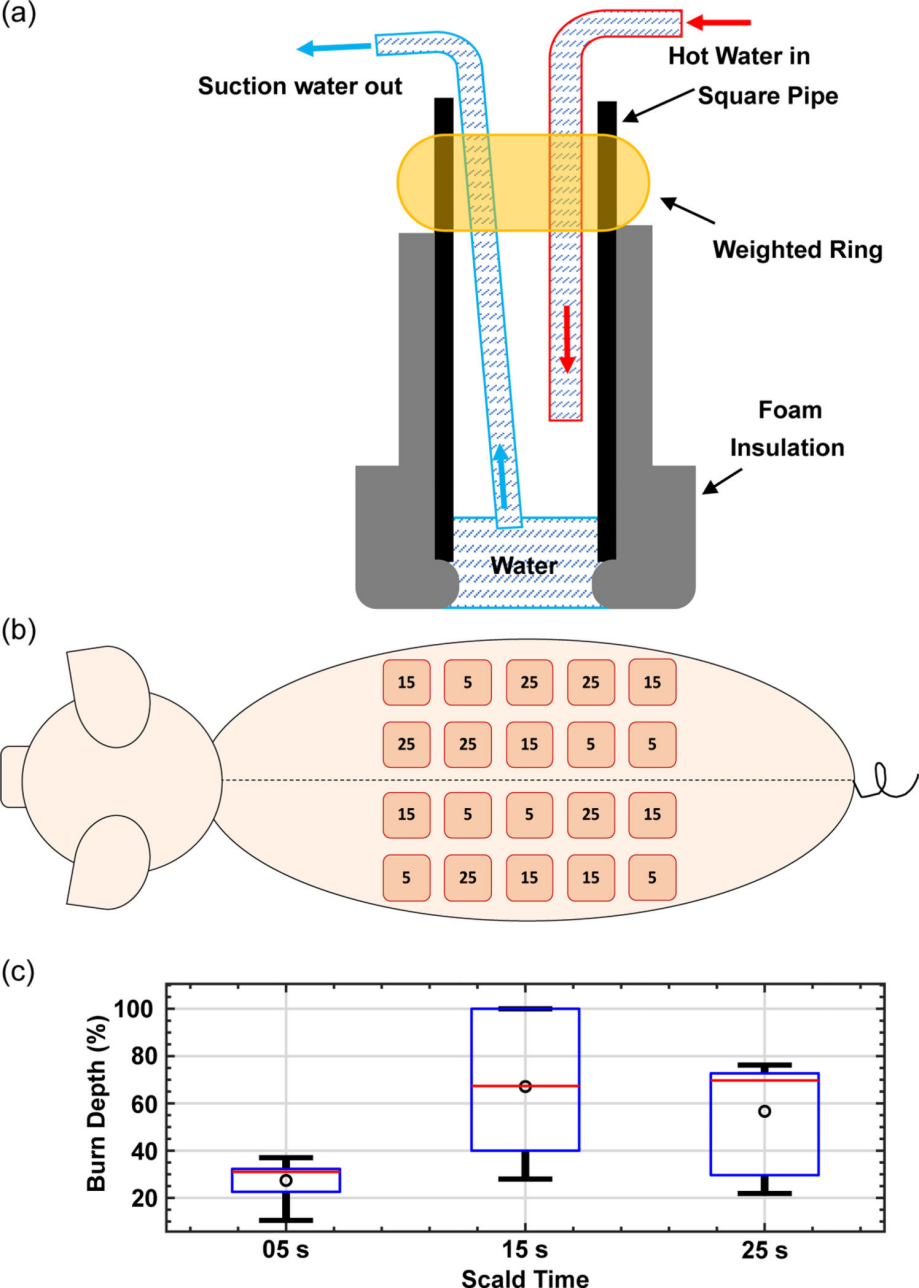
a) The schematic of the scald burn induction device is shown, where 95 °C water would come in direct contact with the skin. b) Scald condition locations are illustrated where the numbers in each circle represent the heat exposure duration in seconds. c) Dermal burn depth percentage (normalized by total dermal thickness) are plotted for each experimental condition, as determined by a histopathologist using Day 4 *H&E*-stained sections.

Scald burns were created on the dorsal side of the pig using the device illustrated in Figure 4a, where a foam-wrapped steel pipe contained a hot water inflow tube and a suction outlet tube.^[40]^ Water was kept at 97 °C using an immersion heating circulator and flowed into the device through high-temperature rubber tubing using the circulation feature on the immersion heating device. Water temperature dropped by ≈2 °C as it flowed through the tubing for a final temperature of 95 °C at the surface of the skin. Hot water was removed from the device using a vacuum pump (Adafruit Industries, New York, NY, USA) attached to a high-density polyethylene (HDPE) vacuum flask. Varied burn severities were created by exposure to 95 °C water for 5, 15, and 25 s, using a 20 × 20 mm^2^ square-shaped pipe in the scald device. Imaging on Day 0 was performed ≈1 h after burn induction. Subsequent imaging sessions on Days 1–4 were performed at the same time each day.

Burns were systematically distributed (*n* = 20 per animal) to minimize variation due to the dorsal location. Burns were separated by ≈4 cm to ensure that each burn was unaffected by the surrounding injuries. The 4 cm distance also provided sufficient area for the THz-TDSI scanner to be placed without interfering with adjacent burns. As illustrated in Figure 4b, an equal number of burns in each experimental arm are created in cranial and caudal sections, in proximal and distal locations to the spine. Based on the size of the animal and previous scald models,^[40]^ the 5-s exposure condition was selected to create a superficial burn, whereas the 25-s condition was intended to create a full-thickness burn. The 15-second condition was selected to create partial-thickness burns that would either progress into a full-thickness burn or continue the healing process by Day 4 as a superficial partial-thickness injury. This dynamic progression effect in the 15-s burns can be seen in Figure 4c by the large variance in the burn depth compared to the 5- and 25-s conditions. The histological assessment of the 15 s burn category on Day 4, shown in Figure 4c, resulted in many severe burns. This increased burn depth is attributed to anatomical variations in the skin thickness, and deeper injury by means of initial damage or wound progression.^[50]^ It is important to note that the statistical analysis of the THz data was not based on severity conditions (i.e., 5, 15, and 25 s groups). Rather, it was based on the histological assessment of the burn depth, as described in the following section.

### Histology:

**Figure 5.**
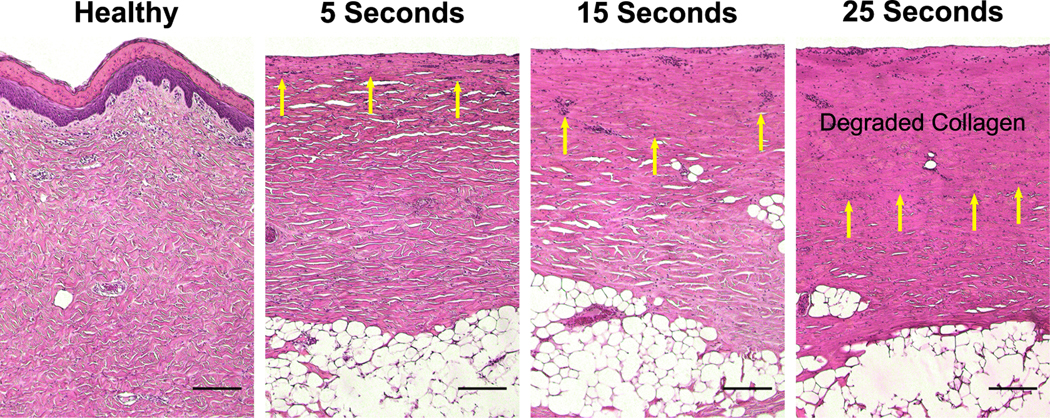
Images of H&E-stained skin samples are shown for each scald condition. Images were captured using a Nikon mm-60 microscope (Nikon Instruments Inc. Tokyo, Japan) with a 5 ×objective lens. Yellow arrows indicate cells marked by nuclear pyknosis. (Scale bar = 0.2 mm).

At the conclusion of the study, a 50 × 50 mm^2^ tissue block that included perilesional and burned skin was excised and fixed in formalin for 24 h before storage in alcohol. Punch biopsies (4 mm diameter) were used to obtain tissue samples from various locations and stained with hematoxylin and eosin (H&E). Histological examples of each scald condition are shown in Figure 5. Blinded evaluation was performed by a board-certified histopathologist. The burn depth was assessed by measuring the deepest point of injury, characterized by microvascular occlusion, collagen discoloration, follicular cell necrosis, mesenchymal cell necrosis, nuclear pyknosis of endothelial and interstitial cells, and adipocyte necrosis for very deep burns.^[51,52]^ The injury depth was then divided by the total dermal thickness and multiplied by 100 to calculate the percent burn depth. Histological sampling was deliberately performed on Day 4, when the burn wound conversion process has been completed.^[53]^ Typically, Day 4 is also the period when physicians wait to determine the true depth of a burn.^[50]^

### Handheld THz-TDSI Scanner:

THz radiation was generated and detected using PCAs as part of an Asynchronous Optical Sampling (ASOPS, Menlo Systems Inc, Newton, NJ) THz-TDS system. The ASOPS system uses two 1560 nm femtosecond lasers, one used to pump an InGaAs/InAlAs THz PCA emitter and the other to probe a LT InGaAs/ InAlAs THz PCA detector. Each laser is set to a slightly offset repetition rate to sample the probe pulse in the time-domain. This is beneficial because, rather than sampling the time-domain (TD) signal in the conventional method that utilizes a motorized stage delay line, the sampling is performed electronically for much faster data acquisition time while maintaining a high signal to noise ratio. The laser repetition rate was set to 100 MHz, with the sampling rate set by the ASOPS difference frequency equal to 100 Hz, which resulted in measurement of a full THz time-domain signal every 0.01 s.

**Figure 6.**
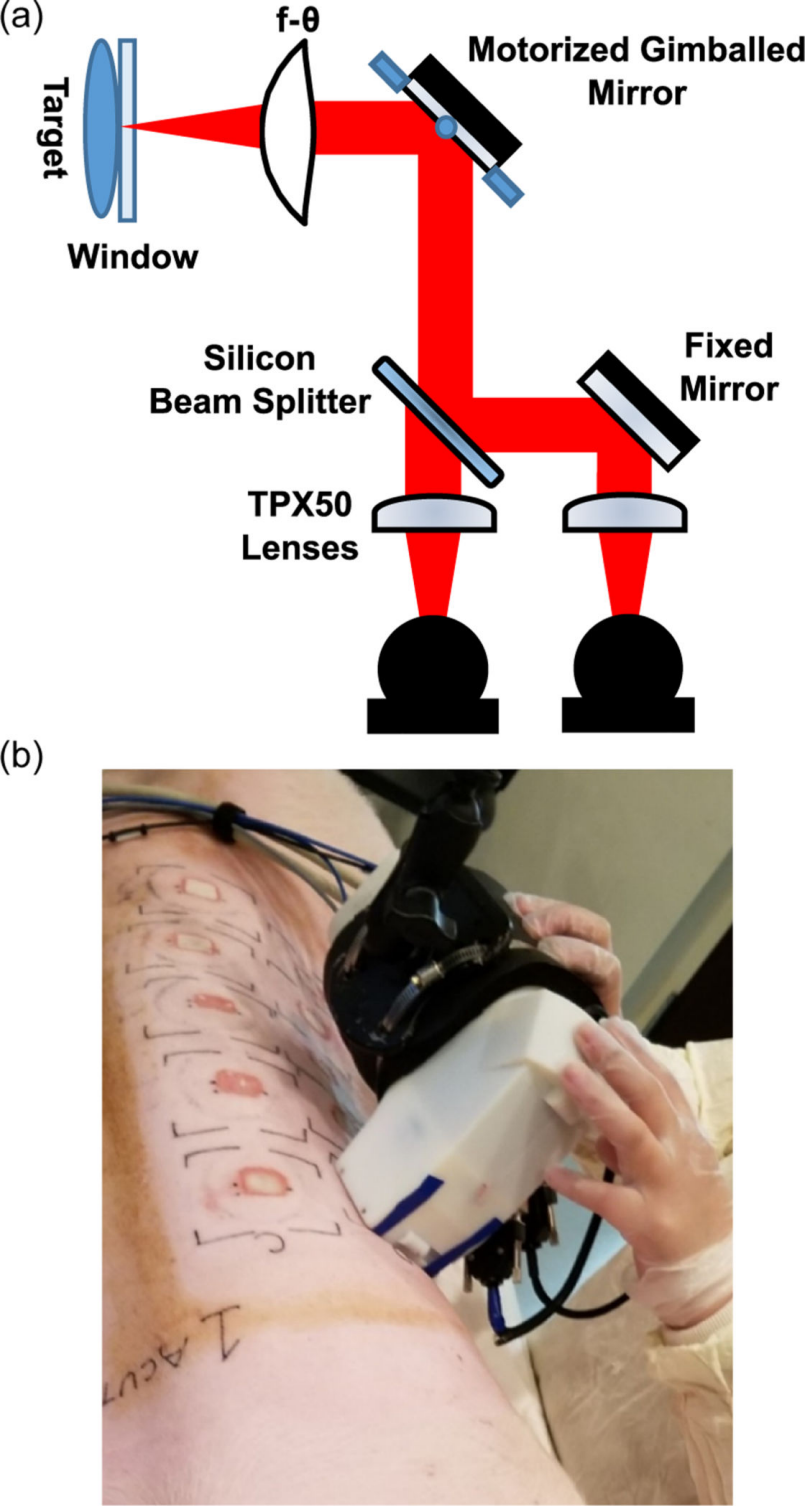
a) The optical schematic for the THz PHASR scanner. b) A visual image of the handheld scanner in use.

THz scans were performed using a Portable HAndheld Spectral Reflection (PHASR) scanner.^[37,54]^ The design of the PHASR scanner, shown in Figure 6a, features a custom HDPE *f*-θ lens and a mirror mounted on a motorized 2-axis gimbal stage (T-OMG, Zaber Technologies Inc. Vancouver, BC, Canada) in a telecentric configuration.^[37,38,54]^ The telecentric nature of the scanner design is ideal for in vivo imaging because it consists of a single beam-steering mirror and ensures a normal angle of incidence over the entire FOV. This, in turn, allows for a collocated emitter/detector geometry. The device also contains a removable fused-silica imaging window in applications where self-calibration is needed or a sample is not perfectly flat. The 12 × 19mm^2^ area can be scanned in ≈1 min with a depth of focus much greater than the penetration depth of the THz beam.^[38]^ Furthermore, the scanner housing allows for an “alignment-free” operation and maintains a constant imaging resolution over the entire FOV. Figure 6b shows the handheld THz scanner in an operating theatre during imaging of porcine scald burns. The design of the PHASR scanner was crucial for this in vivo porcine study because large animals cannot be raster scanned easily. Additionally, their physiological movements, such as respiration, could cause significant imaging artifacts and potential misalignments. Using the PHASR instrument allowed for the movement of the scanning optics in tandem with any animal movements, similar to a portable ultrasound scanner, and therefore enabled an alignment-free and fast imaging operation.

**Figure 7.**
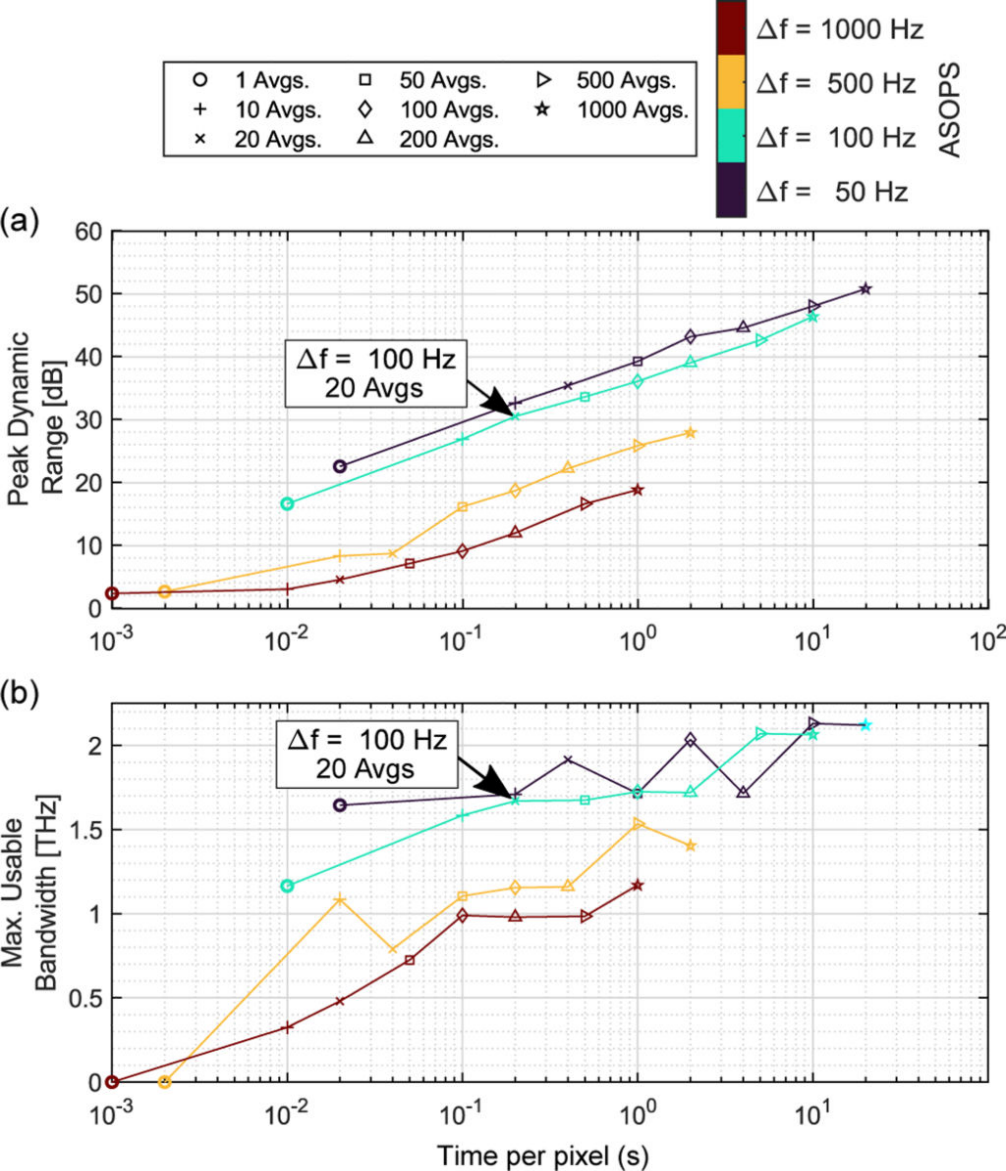
a) Peak dynamic range shows a linear relationship with acquisition time per pixel. Values for peak dynamic range were calculated as the ratio of the maximum frequency domain amplitude to the noise floor level. b) The calculated bandwidth shows a monotonically increasing relationship with the data acquisition time or increasing the number of averages. Marker shapes correspond to the number of averages and marker colors represent the ASOPS difference frequency. Large black arrows represent the optimal operation parameters selected for this study (Δ*f* = 100 Hz, 20 Avgs).

Another feature of our THz PHASR scanner is the use of the ASOPS THz-TDS acquisition technique.^[55]^ The use of ASOPS technique not only eliminates the need for a mechanical delay line, it affords the user the flexibility to easily adjust the scan parameters to optimize performance metrics such as scan speed, dynamic range, and bandwidth. Figure 7 shows the effect of difference frequency tuning and data acquisition time per pixel (i.e., trace averaging) on the peak dynamic range and measurement bandwidth. The black arrow in Figure 7a,b shows the optimum data acquisition parameters selected for this porcine imaging study. The colors of the markers represent the difference frequency between the repetition rates of the two ultrafast lasers in the ASOPS system. The performance values shown in Figure 7a,b were calculated based on reference measurements using a flat mirror. Illustrated in Figure 7a, increasing the number of TDS trace averages, or time per pixel, results in a higher peak dynamic range. Peak dynamic range was calculated as the ratio of maximum reflectivity in the frequency domain amplitude to the noise floor level. In Figure 7b, the bandwidth is calculated as the frequency at which the dynamic range first falls below 0.5 dB. When this frequency point was close to the water vapor absorption lines,^[56]^ the maximum usable bandwidth values deviated from the apparent trend in Figure 7b. While increasing the number of averages would result in higher dynamic range, we chose the setting shown by the black arrows (Δ*f* = 100 Hz, 20 averages per pixel) to achieve a practical acquisition time and a good dynamic range simultaneously. Similar to the dynamic range, Figure 7b shows that the calculated bandwidth has a monotonically increasing relationship with the number of averages and acquisition time.

Further details about the THz handheld scanner can be found in Table 1. When compared with alternative advanced optical imaging techniques such as optical coherence tomography or photoacoustic microscopy, THz systems can scan a larger FOV while maintaining subsecond full spectroscopic acquisition times. Additionally, the THz-TDSI method directly measures the optical properties of the wound, while techniques such as laser doppler imaging and thermography rely exclusively on physiological phenomenon, i.e., perfusion, as a contrast mechanism.

### Signal Processing:

**Figure 8.**
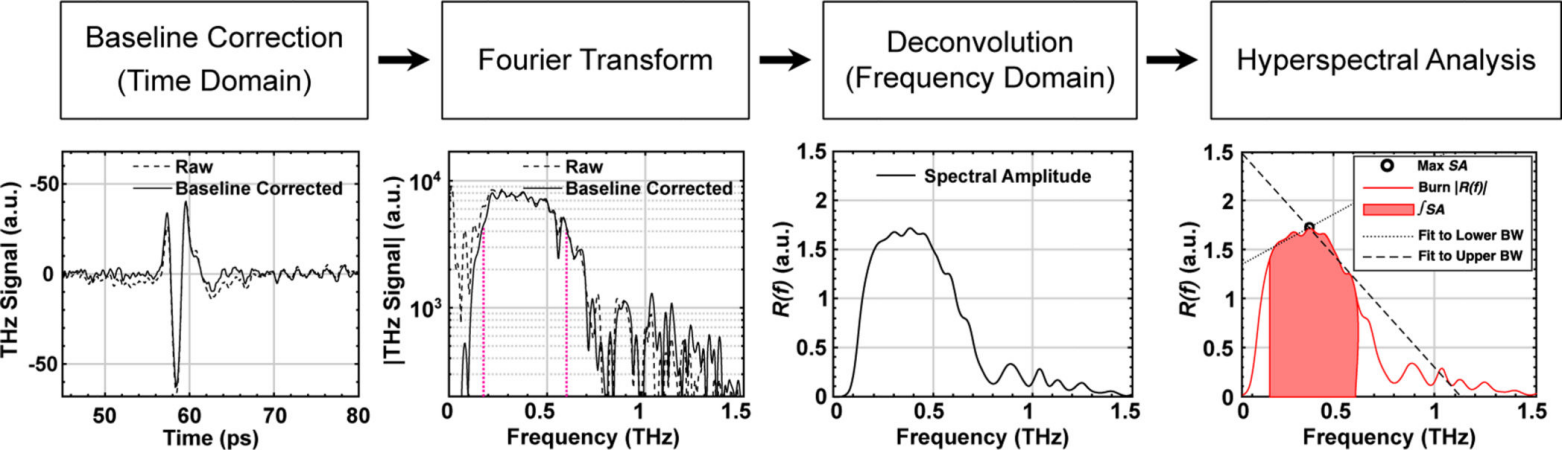
The signal processing flowchart is shown with selected steps depicted below and noted where analysis is performed in the time or frequency domain. Vertical magenta dotted lines represent the *f*_lower_ and the *f*_upper_.

The complete signal processing steps are outlined by the flowchart shown in Figure 8. The raw TD signal in each pixel was first baseline corrected^[57]^ to remove any inherent signal artifacts. A Gaussian bandpass filter, containing signal in our usable BW (0.05–2.5 THz pass band), along with a zero-padded split-Blackman window was applied to the baseline-corrected TD signal. The complex Fourier-transformed representation of the signal, defined as E_samp_ *(f)*, was deconvolved by a reference signal, E_ref_ *(f)*, with the same signal processing steps as the sample. To minimize the impact of noise where the signal-to-noise ratio of the spectra is poor, a Wiener deconvolution algorithm was implemented, where the deconvolved spectral amplitude, *R (f)*, is given by^[58]^

(3) $$R(f)=E_{\text{samp}}(f)\frac{E_{\text{ref}}^{*}(f)}{E_{\text{ref}}(f)E_{\text{ref}}^{*}(f)+\alpha <E>\left[E_{\text{ref}}(f)E_{\text{ref}}^{*}(f)\right]}$$

The Wiener deconvolution implementation uses a Tikhonov Regularization method,^[59]^ where < *E* > is the expected value of the electric field, and α is the regularization constant.

## Figures and Tables

**Table 1.** Specifications for the handheld THz-TDSI scanner developed for burn diagnostic imaging.

Spatial resolution [mm]	Depth of focus [mm]	FOV [mm^2^]	Scan time	Pixel size [mm]	THz intensity [mW cm^−2^]
0.76	9.55	up to 12 × 19	≥0.25 s/pixel	1	≈<0.37
